# *TTS Mapping: *integrative WEB tool for analysis of triplex formation target DNA Sequences, G-quadruplets and non-protein coding regulatory DNA elements in the human genome

**DOI:** 10.1186/1471-2164-10-S3-S9

**Published:** 2009-12-03

**Authors:** Piroon Jenjaroenpun, Vladimir A Kuznetsov

**Affiliations:** 1 Department of Genome and Gene Expression Data Analysis, Bioinformatics Institute, 30 Biopolis str #07-01, Singapore, 138671

## Abstract

**Background:**

DNA triplexes can naturally occur, co-localize and interact with many other regulatory DNA elements (e.g. G-quadruplex (G4) DNA motifs), specific DNA-binding proteins (e.g. transcription factors (TFs)), and micro-RNA (miRNA) precursors. Specific genome localizations of triplex target DNA sites (TTSs) may cause abnormalities in a double-helix DNA structure and can be directly involved in some human diseases. However, genome localization of specific TTSs, their interconnection with regulatory DNA elements and physiological roles in a cell are poor defined. Therefore, it is important to identify comprehensive and reliable catalogue of specific potential TTSs (pTTSs) and their co-localization patterns with other regulatory DNA elements in the human genome.

**Results:**

"*TTS mapping" *database is a web-based search engine developed here, which is aimed to find and annotate pTTSs within a region of interest of the human genome. The engine provides descriptive statistics of pTTSs in a given region and its sequence context. Different annotation tracks of TTS-overlapping gene region(s), G4 motifs, CpG Island, miRNA precursors, miRNA targets, transcription factor binding sites (TFBSs), Single Nucleotide Polymorphisms (SNPs), small nucleolar RNAs (snoRNA), and repeat elements are also mapped based onto a sequence location provided by UCSC genome browser, G4 database http://www.quadruplex.org and several other datasets. The results pages provide links to UCSC genome browser annotation tracks and relative DBs. BLASTN program was included to check the uniqueness of a given pTTS in the human genome. Recombination- and mutation-prone genes (e.g. *EVI-1*, *MYC*) were found to be significantly enriched by TTSs and multiple co-occurring with our regulatory DNA elements. *TTS mapping *reveals that a high-complementary and evolutionarily conserved polypurine and polypyrimidine DNA sequence pair linked by a non-conserved short DNA sequence can form miR-483 transcribed from intron 2 of *IGF2 *gene and bound double-strand nucleic acid TTSs forming natural triplex structures.

**Conclusion:**

*TTS mapping *provides comprehensive visual and analytical tools to help users to find pTTSs, G-quadruplets and other regulatory DNA elements in various genome regions. *TTS Mapping *not only provides sequence visualization and statistical information, but also integrates knowledge about co-localization TTS with various DNA elements and facilitates that data analysis. In particular, *TTS Mapping *reveals complex structural-functional regulatory module of gene *IGF2 *including TF MZF1 binding site and ncRNA precursor mir-483 formed by the high-complementary and evolutionarily conserved polypurine- and polypyrimidine-rich DNA pair. Such ncRNAs capable of forming helical triplex structures with a polypurine strand of a nucleic acid duplexes (DNA or RNA) via Hoogsteen or reverse Hoogsteen hydrogen bonds.  Our web tool could be used to discover biologically meaningful genome modules and to optimize experimental design of anti-gene treatment.

## Introduction

**Figure 1:**
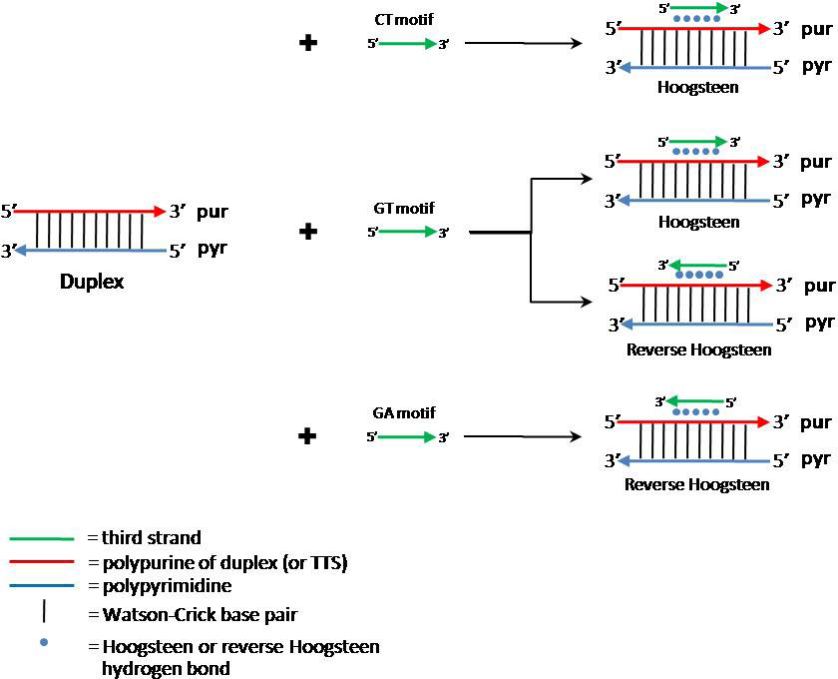
**Schema of three triplex motifs bind to TTS by different orientation**. This schema shows three triplex motifs of third sequence strand which can form triplex structure with polypurine of duplex (or TTS) via Hoogsteen or reverse Hoogsteen hydrogen bond. In CT motif, the third strand is parallel with TTS by forming Hoogsteen hydrogen bond. In the GT motif, the third strand can be either anti-parallel to the third strand by forming reverse Hoogsteen or parallel by forming Hoogsteen hydrogen bond. In GA motif, the third strand is anti-parallel with TTS by forming.

It is known that nucleic acids are flexible molecules that can exist in one of numerous spatial and temporal conformations. In particular, at normal physiological conditions DNA and RNA are both capable of forming helical structures containing some specific genome regions three strands. The existence of triple-stranded nucleic acids, usually named 'triplexes', was first demonstrated by Felsenfeld and collaborators [1]. This conformation is obtained by the insertion of a third strand inside the major groove of a double-stranded (duplex) nucleic acid (Figure 1). Triplexes can contain DNA only, RNA only or the mixture of both, and they are stabilized by the Hoogsteen hydrogen bonds. The most stable triplex conformations are those involving DNA alone or an RNA sequence entering the major groove of a DNA duplex. Among triplexes, the most stable triads are those involving a protonated cytosine (C+) forming C+*GC triad and T*AT nucleotides (the asterisks mark the nucleotides on the third strand). Notably, these sequences are overrepresented in all eukaryotic genomes, as well as in eukaryotic viruses [2,3]. It is also possible to have a triplex formation without homopurine stretches, and these triplexes seem to be as stable as all others, at least, under certain conditions in vitro. Figure 1 shows schematically three possible triplex motifs bound to TTS in different orientation. This schema shows how the third strand of a nucleic acid can form a triplex structure with a polypurine strand of a nucleic acid duplex (or TTS) via Hoogsteen or reverse Hoogsteen hydrogen bond.

Although triplexes have been well characterized in vitro and their role is studying in clinical trials, their biological significance in living organisms is still under discussion [2,4]. It was demonstrated that once formed, these structures can frequently cause down-regulation and sometimes up-regulation of gene expression revealing their potential role in gene expression control and suggesting their application in gene therapy [2,3]. These structures are also able to impair DNA polymerization, and can influence DNA recombination and repair [5]. Triplexes might also have a role in chromatin organization of both interphase nuclei and mitotic chromosomes [6]. Recently, it was demonstrated that a triple stranded pseudo-knot is a conserved essential element of telomerase RNA, for instance in humans [7]. A number of proteins able to bind triplexes have been identified [8]. TTS sequences are common in mammalian genes. Most annotated protein-coding genes in human and other mammalian genomes contain, at least, one unique and high-affinity TTS in their (putative) promoters and/or transcribed regions [3,9].

RNAs are integral components of chromosomes and contribute to their structural organization and the functions of various chromosome regions [10]. Naturally occurring small interference RNAS (siRNAs) and closely related class of non-coding micro-RNAs (miRNAs) could play an essential regulatory role in eukaryotic gene expression and cell function [10,11]. It is now becoming apparent that chromatin architecture and epigenetic memory can be regulated by ncRNA-directed processes, although their exact mechanisms are yet to be understood [10]. It was shown that specific ncRNAs are able to form natural triplexes (purine-purine-pyrimidine triplex structure called H-DNA-DNA-RNA form) with major promoter regions of DHFR gene and switch alternative transcriptional isoforms of genes [11]. miRNA-based anti-gene therapy is now considered as a very promising tool for modulation of gene expression and cell phenotype. However, chromosome co-localization and functional relationships between miRNAs and other types of ncRNAs and TTSs have not been systematically studied.

Certain guanine-rich sequences can often co-localized with TTSs. Certain guanine-rich sequences can fold spontaneously into four stranded DNA structure known as G4 DNA motifs [12]. The structure of G4, which comprises stacked G-tetrads, has a square planar arrangement of four guanine bases stabilized by Hoogsteen GG pairing. It is extremely stable under physiological conditions. Recent studies have demonstrated that the G4 DNA structures formed in regulatory regions (e.g. promoters) could be overlapped with pTTSs and regulate gene expression [12,13]. A well-known example of such regulation is the repressive effect of G4 DNA on the transcription of human *MYC *gene [14]. The transcriptional activity of *MYC *gene is reduced considerably when a parallel G4 DNA formed in the nuclease hypersensitive element III1 upstream of the P1 promoter is stabilized by the G4 ligand TMPyP4 [14]. However, relatively little is known about the detailed molecular mechanism by which G4 DNA influences genome properties. Consecutive guanine structures and guanine content may attribute the high binding affinity of TFOs to their targets [15,16]. Therefore, TTSs including G4 might be perspective genome elements for development novel triplex strategies.

It has been demonstrated in vitro and in vivo that TFOs targeting promoter regions and genic regions of oncogenes and other disease-related genes can diminish the expression of genes, prohibit cell proliferation, and induce apoptosis in the cells [16]. These results also suggest that the effects of TFOs on endogenious gene expression may depend on the specific chromatin structure of specifically addressed gene. In particular, status of nucleosome occupancy might be considered as the factor of efficiency of triplex formation [16-18].

Two TTS WEB resources and search tools of pTTSs in the human genome have been proposed till now. For the first one, TRACTs [19] software, the input is the gene sequence, additionally, the starting and the ending positions of each exon and each intron. The input for this tool can be generated from parsing annotated sequences. The specificity of the input DNA sequence and the annotation of the sequence without explicit intron and exon description of the annotated gene is not reported by TRACTs. The second, TFO target sites tool reported by Gaddis in 2006 [20], has also provide information about TTS sequences for annotated gene (chosen by several annotated Ids). The pTTSs in gene-flanking regions can also be optimized. The both tools use different criteria of identification of pTTSs. The both tools provide a user with information about TTS sequence length and gene region locations. However, the information about TTS within intergenic and intragenic regions of a given protein-coding gene, and any freely selected region on a chromosome might be also important for many reasons, but such opportunities are not available through these two bioinformatics tools. In the context of discovery and analysis of complex genome regulatory elements and genome architectures, it might also be important to study co-localization patterns of natural TTSs, G4 motifs, precursors of non-coding RNAs (ncRNAs), ncRNA targets and other regulatory elements. However, that kind of analysis has not been carried out yet.

In this work we identify and annotate highly-specific pTTSs in any region of human genome and integrate this information with G4 dataset and several USCS genome browser [21] annotation tracks of reported genes, gene models, mRNAs and ESTs. We also provide a multi-track view of the sequences together with several key DNA regulatory elements, including CpG islands, transcription factor (TF) binding sites (BS), single nucleotide polymorphisms (SNPs), nucleosome occupancy profile, repeat sites, and ncRNA precursors and ncRNA gene targets.

## Results

### *TTS mapping *tools features

**Figure 2:**
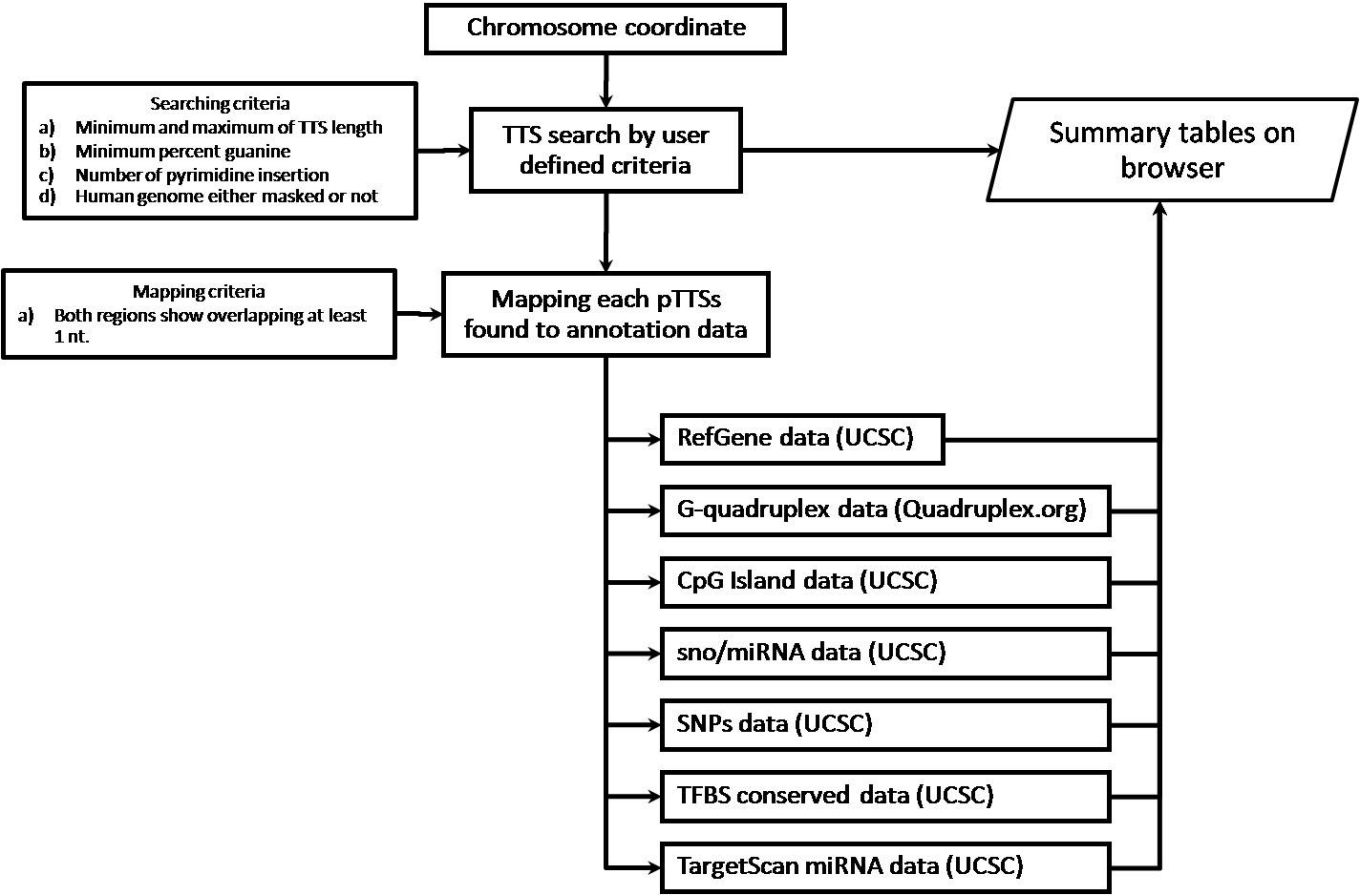
**Schema of data flow in *TTS mapping***. A chromosome coordinate is used to initiate a search of pTTSs in a given region and map pTTSs to several annotation tracks. The pTTSs search parameters should be provided by user or by default. The results of the search are reported in a summary table and also forward to map the given pTTS to selected annotation tracks. After mapping all pTTSs to available annotation tracks, the number of found annotation tracks, the number of the annotation tracks overlapped with these pTTSs, and number of the pTTSs overlapped with the annotation tracks are reported to summary table.

**Figure 3:**
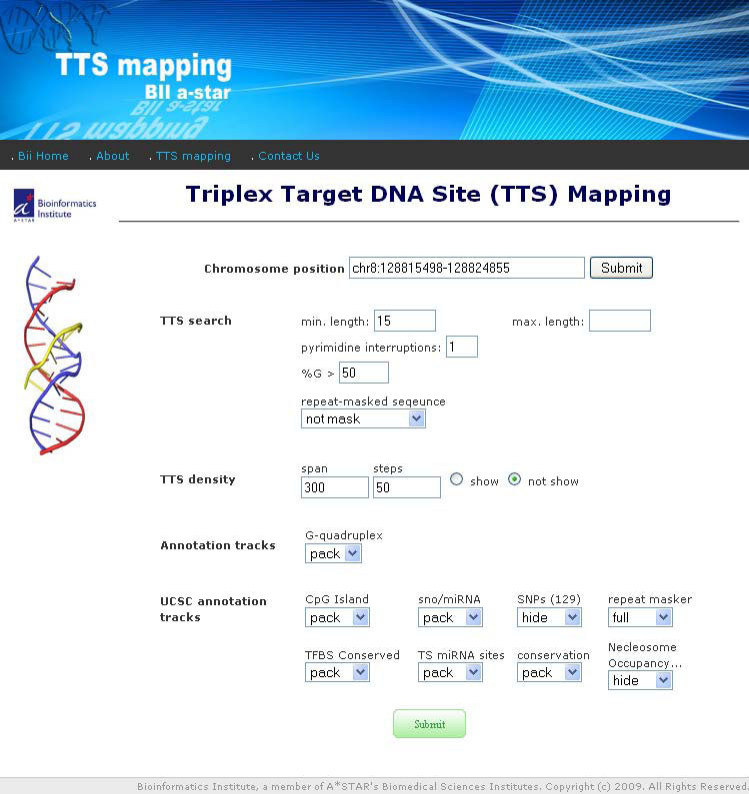
**The interface page of *TTS mapping *tools**. To run the program, user has to define chromosome region and parameters for TTS search. "TTS density" option could be used to show TTS density in the running window with a given span and window moving step.

The schema of data flow in the *TTS mapping *tools is presented on Figure 2 and described in the Methods section. We have developed a web-based search tool to find polypurine sequences, which could be pTTSs, and to map them to human genome along with annotation tracks from a number of other databases. The web interface allows a user to modify various search parameters (see Figure 3). The search parameters (chromosome position, minimum and maximum length of TTS, number of pyrimidine insertion, percentage of guanine composition, and repeat masking) defined by user are used as criteria for TTS searching. The default TTS search parameters were set with minimum of TTS length of 15-nt, minimum percent G composition of 50%, and number of pyrimidine insertion of 1. To display the TTS density in a given region in UCSC genome browser, the TTS density option (defining window span and moving step) is provided. In addition, the form also allows a user to choose several annotation tracks used for chromosome position characterization and for the annotation of pTTSs-containing genomic regions.

### TTS search parameters

#### TTS length correlates with TTS uniqueness in the human genome

The statistics of pTTSs aligned against the human reference genome (hs18) is studied in this section. The statistical distribution of TTS length demonstrates that relatively short TTS sequences (15-19 nt length) often map many genome regions (Additional File 1, 2). However, a vast majority of TTSs with length larger than 18 nt exhibits unique genome coordinates. Using BLAST algorithm (see Method section), we found the uniquely located pTTSs and counted their abundance. We found 519,971 TTSs in the reference human genome. Table 1 shows that pTTS sequences with length 19-24 nt are most often occurred in the genome (58% (303,789/519,971)) than the short (15-18 nt; 13%) and the long (25-872 nt; 29%) TTSs, respectively.

**Table 1:** A comparison of the frequencies of uniquely mapped pTTSs found in the TTS databases

TTS length		Number of unique pTTSs		Ratio of [B] and [A]
			
		Wu et al. 2007 [A]	TTS mapping [B]	
Short	15-18	441471 (46%)	67292 (13%)	0.152
Medium	19-24	371267 (39%)	303789 (58%)	0.818
Large	25-872	140882 (15%)	148890 (29%)	1.057
all length	15-872	953620 (100%)	519971 (100%)	0.545

We also count the number uniquely located pTTSs identified by Wu et al. [22]. Table 1 shows that the total number of TTSs in our DB contains 55% of TTSs reported by Wu at al. However, this table also shows that short TTSs consist of a major fraction (46%) of the TTS population. The numbers of medium-length (19-24 nt) and long-length uniquely located TTSs in the both databases (DBs) are similar. Thus, our DB exhibits stronger filtration of the short TTSs than Wu et al. DB. This difference could be explained by difference in pTTS alignment criteria and additional filtering procedures related to multiple mapping of the pTTSs reported in [22]. More information regarding a comparison of non-unique and unique sequences in the DBs is presented in Additional file 2.

#### Pyrimidine insertion

Another limitation of triplex binding is pyrimidine insertion in TTS. Only a single pyrimidine insertion can greatly decrease triplex stability [15,23]. Although modified TFOs can overcome the limitation, the insertions still affect triplex stability in these cases [24]. Nevertheless, a few pyrimidine insertions still have to be considered. There are several reports in the literature about the use of modified TFO for an efficient inhibition of gene expression. For example, for the inhibition of *MYC *gene expression it was demonstrated that a 25 nucleotides long TTS with 3 thymine based insertion was required [25,26]. Therefore, our *TTS mapping *keeps a flexibility of search for pTTSs with pyrimidine insertion.

#### G content and TTS length

It was shown that TFOs are able to form stable triplex structures when their length is at least seven nucleotides (nt) [27]. The further studies indicated that TFOs longer than 7 nt can form more specific bonds with their targets than the shorter ones [15,28,29]. Most of high-affinity binding between TFO and TTS requires high G content (>54% of the total nucleotide sequence length) in the TTS sequences [15,23]. However, the experiment of Vekhoff et al. demonstrates that GU-TFOs can form stable triplexes to its target with minimal G composition of 40-50%, depending on TFOs or TTS length and sequence context [30].

The latter three components were used to identify pTTSs, depending on the user's choice. In *TTS mapping *tool, 15 nucleotides is used as the default minimal TTS length, because, even though 15 nucleotides length does not guarantee a strong uniqueness, such sequences may still be interesting if they overlap with transcription factors binding sites or other regulatory elements. The default minimal G content is set to 50%. This value was chosen in order to ensure that triplex structure could be formed and be stable [30].

#### Summary tables of *TTS mapping*

**Figure 4:**
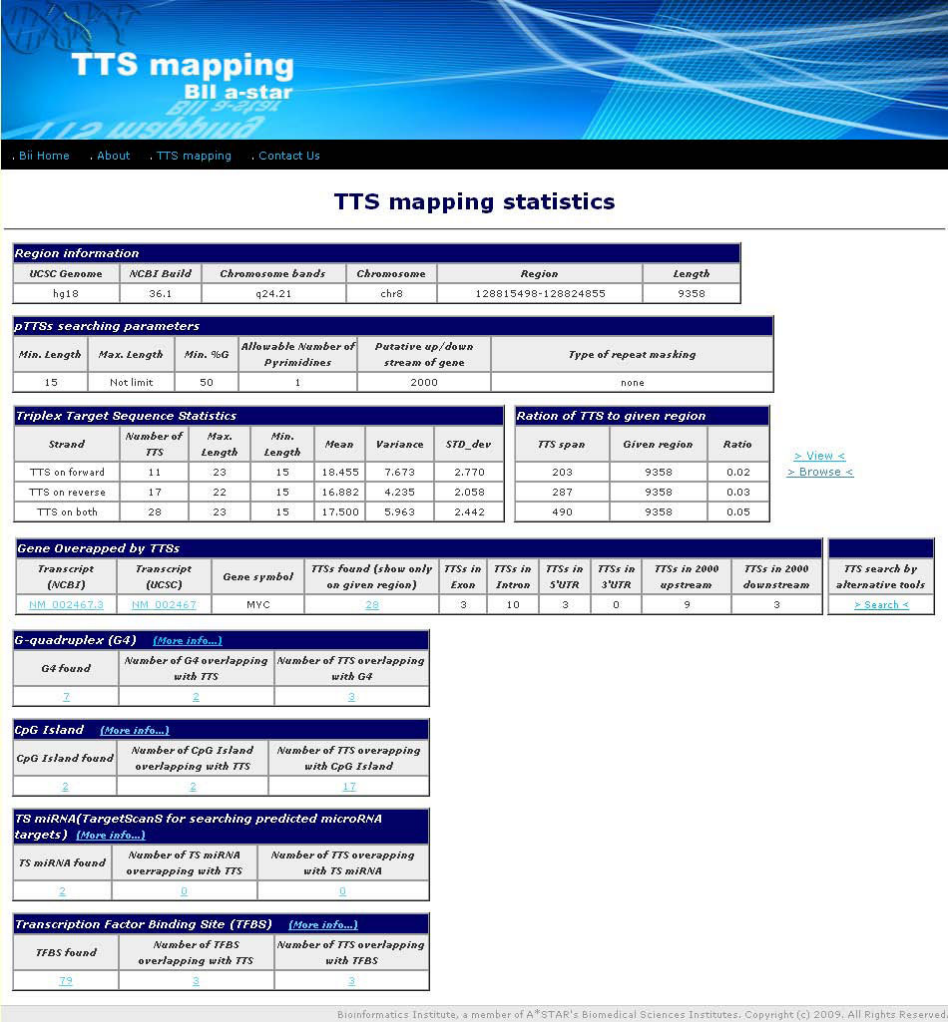
**The summary tables of pTTSs search and mapping in a human genome region**. The results display summary tables of: chromosome position, TTS search parameters, statistics of pTTSs found, the gene appearing in the given chromosome position, and the data of selected annotation tracks found in the chromosome region.

**Figure 5:**
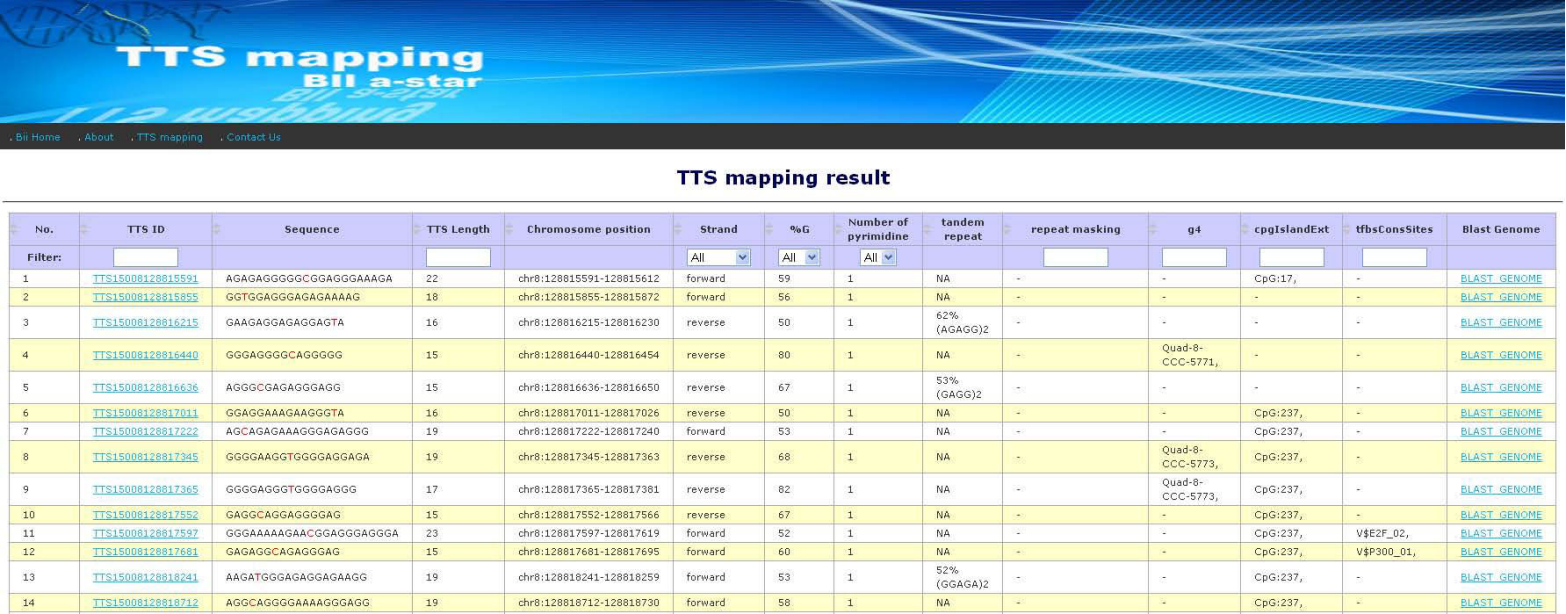
**The list of pTTSs and integrative annotation information**. The list of to TTS ID which describe the descriptive information of each pTTSs and integrate annotation tracks overlapped with pTTSs. The link of TTS ID enable the user to view information about pTTSs in context of the chromosome sequence, tandem repeat inside the pTTSs, the known repeat element overlapping with pTTSs, other annotation tracks overlapped, and the BLAST genome link. The BLAST link is use to view the uniqueness of pTTSs on the human genome.

The results page displays summary tables (Figure 4) in several topics. The first one, the region information table, describes the genome version in UCSC database and NCBI database, chromosome bands, chromosome position, and length. The second one, the table of the pTTSs search parameters, describes the parameters defined by user. The third one, the pTTSs statistics table, describes the number of pTTSs found in the given chromosome position and descriptive statistic for each strand separately and both strands taken together. The user can view the table of the descriptive information and list of annotation tracks overlapped of each pTTS (Figure 5) by clicking at "View" or display the pTTSs on UCSC genome browser by clicking at "Browse" beside the table. The fourth one, the gene table, describes the gene name and transcript identifier (transcript ID), appearing in a given chromosome region, the number of pTTSs overlapping with the gene, and number of pTTSs found in gene-associated locations (exon, intron, 5'UTR, 3'UTR, and 2 kb up/down stream of gene). More details on each pTTS in a gene are provided in the link for the total number of pTTSs (Figure 4). The detailed information about pTTSs in a gene includes: nucleotide sequence, chromosome position, percent G composition, type of sequences (polypurine or polypyrimidine), the distances of pTTS from TSS and from start or end of gene, and gene locations. The fifth one, the set of other summary tables, contains the information about the annotation tracks, which are G4, CpG Island, snoRNA, miRNA, SNPs, TFBS, and miRNA targets. The summary table of annotation tracks describes the number of tracks found, the number of tracks containing pTTSs, and the number of pTTSs located in the tracks. Links in these tables are provided to display the detailed information for each track.

**Figure 6:**
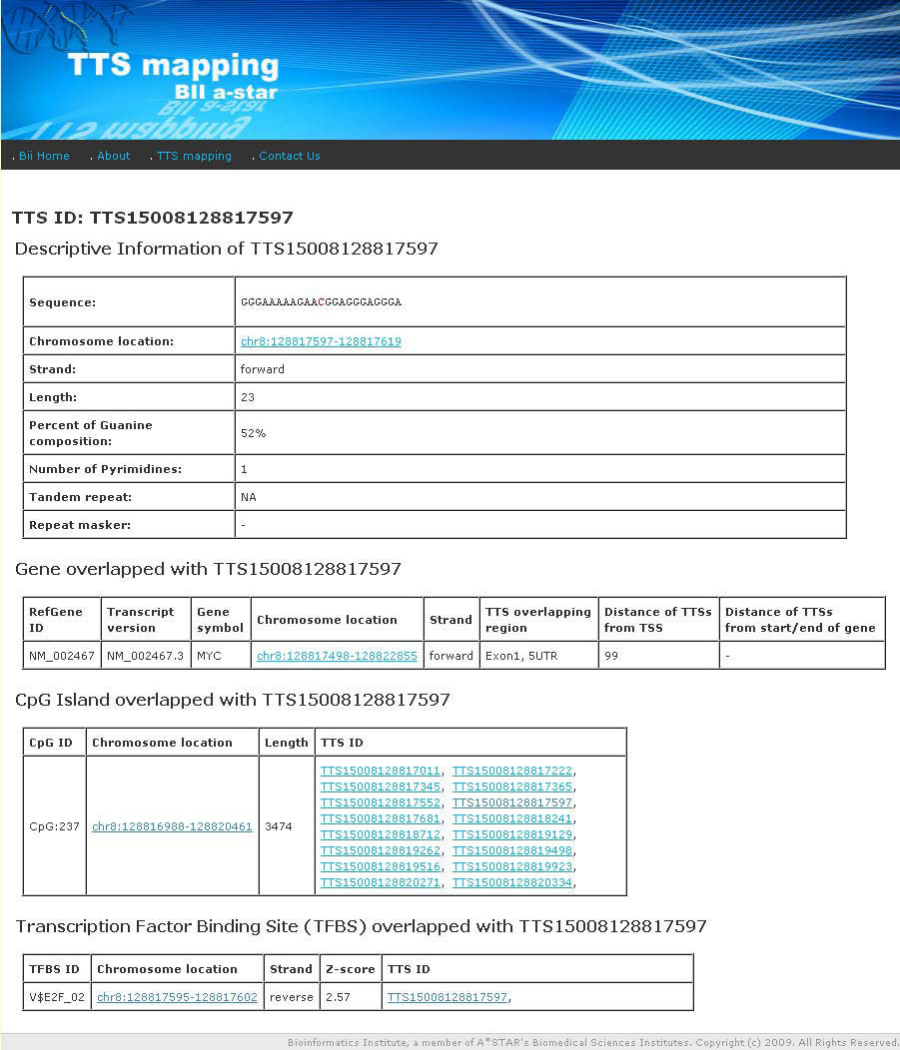
**Result Tables on the properties of pTTS and neighboring annotation information**. Descriptive information about pTTS, neighboring gene region which overlapped with the pTTS and annotation tracks which overlapped with the given pTTS.

**Figure 7:**
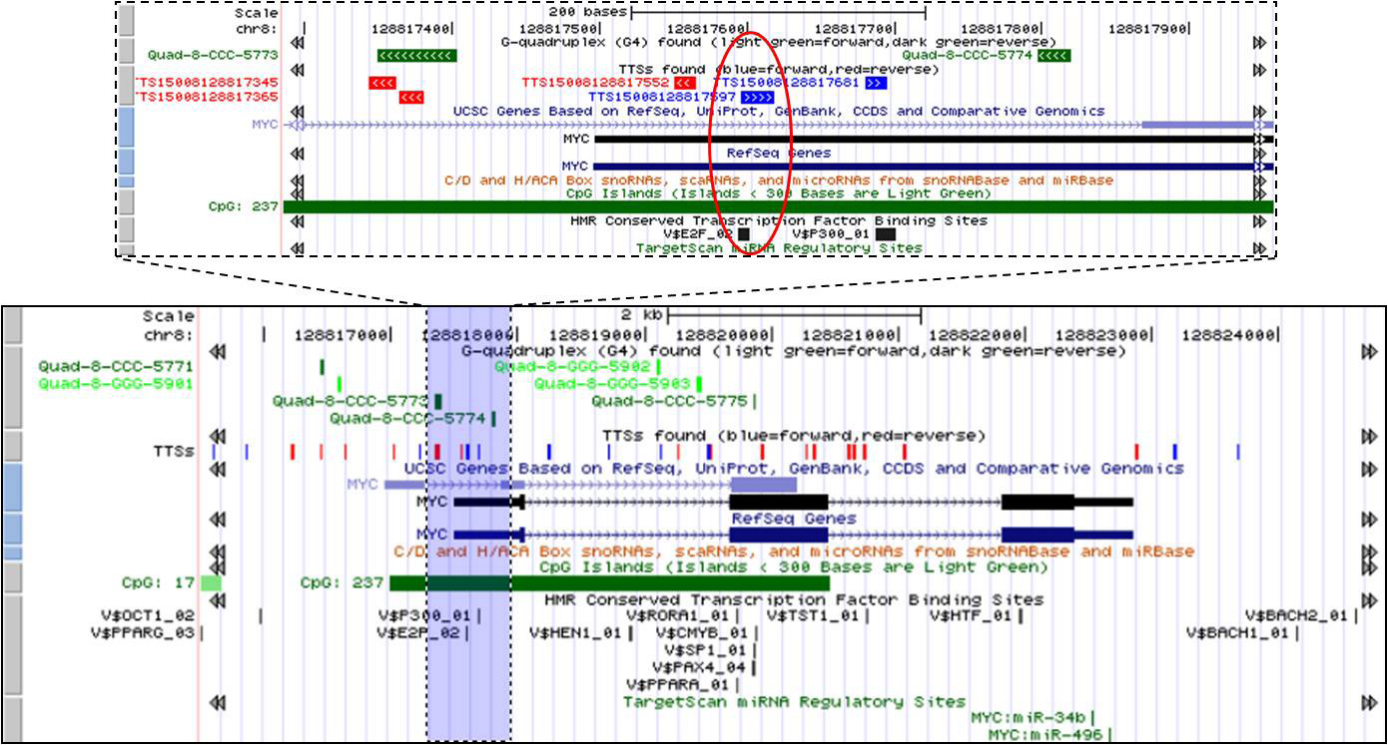
**Mapping of pTTSs and other regulatory elements on *MYC *gene region**. Bottom plot shows detail map of pTTSs and maps other regulatory elements on *MYC *gene region and up plot shows zoomed region and regulatory sequences in the vicinity of start position of *MYC *gene. Red oval in the zoomed region: The uniquely mapped TTS (TTS15008128817597) located in the first exon of *MYC *gene is overlapped with the TFBS V$E2F_02 and with the CpG island. The next downstream TTS (TTS15008128817681) in the fist exon of *MYC *gene is also overlapped with other TFBS V$P300_01. However, for the last pTTS multiple locations in the human genome were found. Two G4 sequences (Quad-8-CCC-5773 and Quad-8-CC-5774) are also shown in a vicinity of start position of *MYC *gene One of this G4 (Quad-8-CCC-5773) is overlapped with pTTS TTS15008128817345 and include other pTTS TTS15008128817365.

**Figure 8:**
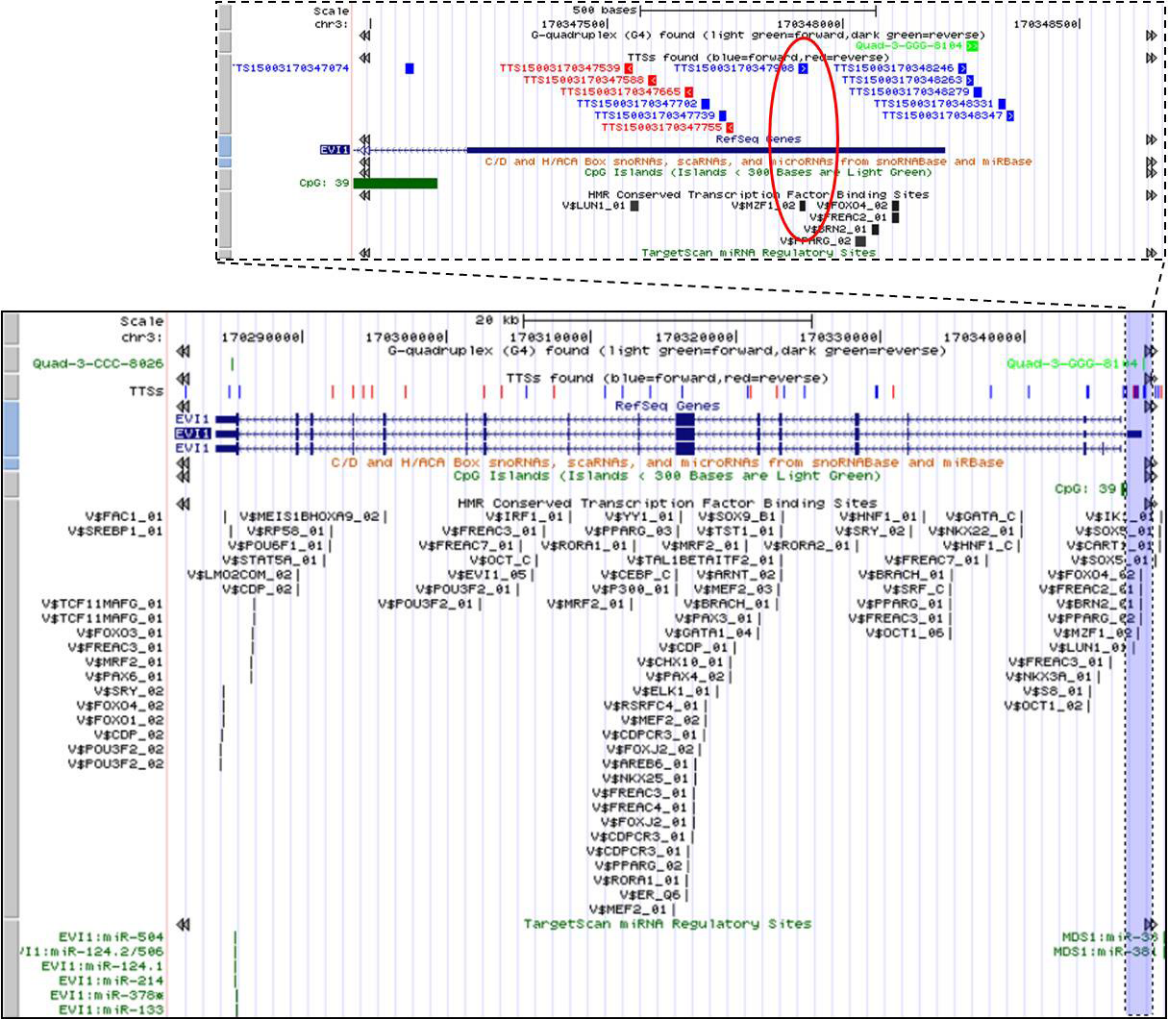
**Mapping of pTTSs and other regulatory elements on *EVI-1 *gene region**. Red oval: the unique pTTS is overlapped with transcription factor binding site.

**Figure 9:**
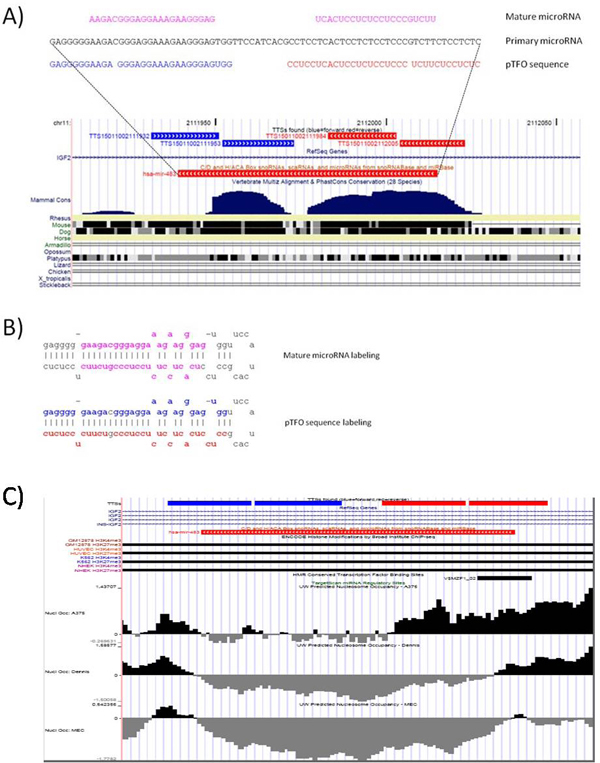
**Mapping of pTTSs and miRNA included into *IGF2 *gene region**. A: Mapping on primary miRNA (hsa-mir-483) and pTFO on the evolutionally conserve intronic region of *IGF2 *gene. B: secondary structure of primary miRNA (hsa-mir-483). Pink color of nucleotide symbol: mature siRNA, blue color of nucleotide symbol: polypurine sequence, red color of nucleotide symbol: polypyrimidine sequence located on the some strand as double helix DNA. One substitution on Y in the R starch is allowed. C: pTTS (TTS15011002112005) includes binding site (TTCCCCTCTCCC) of transcription factor MZF1.

#### List of found pTTSs and integrative annotation information

The descriptive information about pTTSs is listed in a table by TTS ID (in Figure 5). TTS IDs are created consisting of 17 characters in the following order: "TTS", pyrimidine insertion, %G, chromosome, start position of pTTS, for example: "TTS 3 50 08 128815591". Each TTS ID refers to sequence, chromosome position, G composition percent, an overlapping tandem repeat, an overlapping known repeat element overlapped, overlapping annotation tracks overlapped, and a BLAST genome link, which is used to evaluate the uniqueness of pTTSs in human genome. An application for sorting and filtering tables is accessible in column captions. In addition, the user can click on TTS ID to display its descriptive information and details on each overlapping annotation track overlapped.

#### Descriptive statistics table of pTTSs and the genomic regions overlapping with pTTSs

Detailed descriptive information about each pTTS is described in a table represented in Figure 6. Descriptive information table contains pTTS sequence, chromosome position, strand, %G, the number of pyrimidine insertions, tandem repeats, and known overlapping repeat regions. The table also displays overlaps of the pTTS with genes. The detailed information about other overlapping tracks is also displayed when such tracks overlapping are found. A case study of pTTSs found in the genes of transcription factors

It has been shown that TTS could be co-localized with TFBSs in the same putative promoter region [15,23,30]. For insurance, figure 7 shows several pTTSs found by our *TTS mapping *software in the region of *MYC *gene. In zooming region on Figure 7 the start position of *MYC *gene includes the uniquely mapped TTS TTS15008128817597) which overlapped with the TFBS V$E2F_02. The next downstream TTS (TTS15008128817681) is located in the fist exon of *MYC *gene which overlapped also with BS V$P300_01. However, TTS15008128817681 pTTS has multiple locations in the human genome. Additionally, several other annotation tracks including G4, TFBS, miRNA target, and CpG Islands are also displayed on Figure 7. More detailed information about pTTSs and other putative regulatory sequences is presented in the supplementary materials (additional files 3, 4). Using BLAST alignment of pTTS TTS15008128817597 against the human genome we found a unique location of the pTTS. This TTS is overlapped with the TFBS V$E2F_02. Potential importance of TTS TTS15008128817597 for anti-gene therapy has been demonstrated. Inhibition of gene expression via pTTS TTS15008128817597 in vitro was found by several research groups [25,31,32].

In another case study (Figure 8), we identified a pTTS TTS15003170347908 which sequence is unique in the genome and is overlapped with the TFBS V$MZF1_02. MZF1 is TF belonging to the *Krüppel *family of zinc finger proteins, expressed in totipotent hemopoietic cells as well as in myeloid progenitors. MZF1 can act as a tumor/growth suppressor in the hemopoietic compartment [33].

The same pTTS is also located in *EVI-1 *gene isoforms: the first exon of NM_001105078 and in putative 2 Kb-promoter regions of *EVI-1*isoforms NM_001105077 and NM_005241. Detailed information about *EVI-1 *region is presented in supplementary materials (additional files 5, 6, 7, 8). We found no evidences in the literature of possible function or therapeutic applications of this pTTS. Biological importance and clinical significance of these associations requires a further investigation. The presented results suggest that simultaneous mapping of specific regulatory elements (unique pTTS and TFBS) presented on Figure 8 could provide important information regarding new application in targeting *EVI-1 *genes.

#### TTS and G4 co-occurrence

It has been shown that polypurine oligonucleotides can form antiparallel triplexes with G-rich TTSs in promoter regions of c-myb, MYC, Ki-ras (and some other oncogenes) and reduce both the proliferation and colony formation in tumor cells [16,25,34]. These G-rich TTSs could be also overlapped or included in the G4 DNA and/or G4 RNA structures, which probably could modify gene expression due to triplex formation at both the transcription and post-transcription levels [34,35]. We observed many putative promoter regions (around transcription start position) where pTTS and G4 are overlapped or imbedded. The examples of such co-localization events are presented on Figure 7 and Figure 8. Figure 7 shows a map of pTTSs and other regulatory elements on MYC gene region. Table 2 shows detailed information about G4 track overlapped TTS in MYC promoter region generated by link to G4 track presented in the Figure 4. Two G4 sequences (Quad-8-CCC-5773 and Quad-8-CCC-5774) present in a vicinity of start position of MYC gene. One of these G4s, Quad-8-CCC-5773 is overlapped with pTTS TTS15008128817345 and includes other pTTS (TTS15008128817365) (Table 2). Note, G4 Quad-8-CCC-5774 is located in 5'UTR region and does not show overlapping with any TTS (Figure 7). In the case of EVI-1 gene two TTS (TTS15003170348263 and TTS15003170348279) are overlapped with G4 (Quad-3-GGG-8104) in the putative promoter region (Figure 8). These examples suggest a new challenge for systematic study of TTS and G4 interactions and an opportunity for optimization triplex anti-gene therapy.

**Table 2:** G4 and pTTSs overlapping in the *MYC *promoter and MYC intragenic regions pTTSs overlapped with G4s are in bold type.

G4 ID	Sequence	Chr	Start	End	Strand	TSS ID
Quad-8-CCC-5773	**CCCCACCTTCCCC**A**CCCTCCCCACCCTCCCC**ATAAGCGCCCCTCCCGGGTTCCC	Chr8	128817351	128817404	-	TSS15008128817345 TSS15008128817365
Quad-8-CCC-5771	**CCCCCTGCCCCTCCC**ATATTCTCCC	Chr8	128816440	128816464	-	TSS15008128816440

#### TTS can co-occurred in ncRNA precursor

Output of *TTS mapping *exhibits an often occurrence of TTSs in the precursors of small ncRNAs located in the introns of protein coding genes. For instance, figure 9A shows an overlapping between miRNA precursor, hsa-mir-483, located in intron 2 of short isoform of *IGF2 *gene. The *IGF2 *gene encodes a member of the insulin family of polypeptide growth factors that is involved in development and growth. *IGF2 *can stimulate various cellular responses acting as a cell survival factor or mitogenic factor and can also modify metabolism. It is an imprinted gene and is expressed only from the paternally inherited allele. Although *IGF2 *is normally only transcribed from the paternal allele, maternal imprinting is lost in many tumors, leading to biallelic expression of the gene. Disruption of imprinting and the resulting increase in gene dosage have been implicated in tumor transformation in a variety of human tissues [36]. Loss of *IGF2 *imprinting leads to an oncogenic diathesis that enhances the risk for neoplastic transformation. There is a read-through, INS-*IGF2*, which aligns to this gene at the 3' region and to the upstream *INS *gene at the 5' region.

*TTS mapping *found two polypurine pTTSs and two polypyrimidine pTTSs in intron 2 of *IGF2 *(Table 3). These pTTS sequences are located in the evolutionally conserve region of intron 2 of *IGF2*. Note that the Triplex-forming Oligonucleotide Target Sequence Search engine http://spi.mdanderson.org/tfo/about.php lost TTS GTGGGGGAGAGGGGGAAGA in output overlapped gene table. Figure 9B shows secondary structure of miRNA precursor (hsa-mir-483) which includes polypurine and polypyrimidine sequences within a region of the mature siRNA. Interestingly, intron 2 of *IGF2 *contains a large number (9 transcripts) of overlapping transcripts, while in intron 1, intron 3, and intron 4 the number of overlapping transcripts was 0, 2 and 1, respectively. Figure 9B also shows the location of transcript polypurine or polypyrimidine sequences in intron 2 on the secondary structure of the siRNA derived from intron 2. DNA sequence TTCCATCACG, the linker between polypurine (GAGGGGGAAGACGGGAGGAAAGAAGGGAGTGG) and polypyrimidine (CTCTCCTCTTCTGCCCTCCTCTCCTCACTCCTCC) sequences of mir-483 precursor, forms a non-complimentary head of the RNA harping loop (UUCCAUCACG). This linker sequence is not evolutionarily conserved.

**Table 3:** Four TTSs overlapped with mir-483 precursor

TTS ID	Sequence	TTS Length	Chromosome	Start	End	Strand	%G	sno/miRNA ID
TTS15011002111984	GGGAGGAAAGAAGGGAG**T**GG	20	chr11	2111984	2112003	reverse	60	hsa-mir-483
TTS15011002111953	GGGAGGAGAGGAG**T**GAGGAGG	21	chr11	2111953	2111973	forward	67	hsa-mir-483
TTS15011002111932	AAGGG**C**AGGAGAGGAGAAGA	20	chr11	2111932	2111951	forward	50	hsa-mir-483
TTS15011002112005	G**T**GGGGGAGAGGGGGAAGA	19	chr11	2112005	2112023	reverse	68	hsa-mir-483

*TTS mapping *could predict the non-trivial functional links between genome localization of specific evolutionally conserved TTSs and essential regulatory protein-coding genes, natural siRNA precursors and share TF BS in gene promoter regions. For instance, the TTS-rich regions included into intron 2 of *IGF2 *gene covers the mir-483 precursor region. One of TTS includes binding site of TF MZF1. Figure 9C shows that one of four pTTS (TTS15011002112005) includes the binding site (TTCCCCTCTCCC) of TF MZF1. Additionally, TTS mapping exhibits trimethylation of hystone 3 lysine in the position 27 (H3K27met3) and an absent of trimethylation of hystone 3 lysine in position 4 (H3K4met3) of *IGF2 *gene region observed in several cancer cell types, which suggests that transcription of the both *IGF2 *gene and mir-483 is suppressed. We suggest that the TTSs could form triplexes in and TTS-rich regions included into intron 2 of *IGF2 *gene and, thus, TTS-TFO complex might be involve in mechanisms of local epigenetic regulation of repressive heterochromatin directly or/and through the recruitment of specific proteins (for instance, MZF1 and triplex-binding proteins directly interacting with TTSs). Data on negative nucleosome occupancy of evolutionarily conserved TTSs in the mir-483 precursor region (Figure 9), exhibited in USCS viewer, support our hypothesis. Thus, we could suggest that both the *IGF2 *gene and the precursor mir-493 are essential genes (in fetal tissue development and cancer growth) which might be controlled by genetic and epigenetic mechanisms driven by natural TTSs-TFO complexes.

## Discussion

In this work we provided an automatic cartography of specific pTTSs within the human genome and integrated this information with other identified regulatory DNA motifs and sites. Our analysis shows that *TTS mapping *can provide a comprehensive and detailed analysis of integrity TTSs with other genomic regulatory signals essential for understanding of genome stability and gene expression.

We developed a flexible web-based search tools for finding, annotating TFO G-rich TTSs within the human genome and integrating this data with G4 motifs and other regulatory elements, including CpG Island, miRNA precursors, miRNA targets, transcription factor binding sites (TFBSs), Single Nucleotide Polymorphisms (SNPs), small nucleolar RNAs (snoRNA), nucleosome potential and repeat elements. Descriptive information about each genome region, including sequence context overlapping annotated DNA sequence regions and gene regions (e.g. introns and exons) and putative promoter and downstream region is provided. The engine assists the user in finding highly-specific TFO TTS (pure polypurine sequences with length larger than 14 bp) and "moderate specific" TFO TTS (TFO purine target sites with length larger than 14 bp, including 1, 2 or 3 pyrimidine insertions).

Using well studied oncogenes *MYC *and *EVI-1*, which exhibit strong recombination- and mutation-prone functions (which are often associated with human malignancies) as examples, we demonstrated that pTTS, TFBS, and miRNA targets and several other regulatory elements could be synergistically involved in co-modulation of the promoter regions of *MYC *and *EVI-1*.

*TTS mapping *reveals that a high-complementary evolutionarily conserved polypurine and polypyrimidine motif pair linked by the non-conserved short sequence, form miR-483 precursor. Originally, miRNA hsa-miR-483 was located in chromosome 11 and was identified by [37] in fetal liver in human. In addition, miR-483 was annotated in mouse and rat by sequence similarity in mirBase [38,39]. This ncRNA was detected in an automatic scan (composite score 8.6, energy 31.4) and was scored highly as a miRNA by RNAmicro [40]. Hsa-miR-483 was also detected by RNAz but was not in the input set for EvoFold [41]. Predictions of miR-483, in addition to human, rat and mouse, also were made for dog, cow, horse and rabbit http://people.csail.mit.edu/akiezun/miRviewer/mir-483_index.html. So, miR-483 DNA precursor is strongly conserved across many mammals and contains a high-complementary polypurine and polypyrimidine motif pair linked by a short low-conserved sequence forming hairpin loop of secondary structure of this ncRNA. miRNA can be co-transcribed by RNAPol-II with protein-coding gene *IGF2 *in which miRNA precursor is embedded. We suggest that R-Y TTS pair forming a natural DNA-RNA triplex within precursor mir-483 transcribing from the second intron of *IGF2 *gene might be an important target for endogenous regulation of the functions of this ncRNA in the human cells.

BLAST aligning of pTTS with the reference human genome revealed the unique overlapping of pTTS with TFBSs (Figure 8 and Figure 9). Inhibition of gene expression via TFOs forming the triplexes with such TTSs was demonstrated by several research groups [25,31,32]. In particular, it was shown that triplex-forming oligonucleotides can bind with a high specificity to the *MYC *promoter in HeLa cells, thereby reducing *MYC *mRNA levels in the cells [25,31,32].

Figure 8 and Figure 9C shows that pTTSs TTS15003170347908 and TTS15011002112005 include common anti-sense sequence of BSs of TF MZF1 (having TFBS TTCCCCTCTCCC) which could mediate a control of transcription of genes *IGF2 *and *EVI-1*(Figure 8). These examples suggest that transcription of two (or more) essential genes could be specifically directed via a single highly-homologous TTS. Other DNA-binding proteins (e.g. nuclear protein bound the TTS in promoter region of Ki-ras and MYC genes [14,34]) can induce site-specific modifications of genome DNA structures and, finally, changes of gene expression. Additionally, silencing mechanism(s) of the gene expression could be regulated by some proteins directly interacting with pyrimidine-rich motif (s) of RNAs. For instance, CRD-BP/IMP1 is an oncofetal RNA-binding protein which, via short pyrimidine-reach binding sites, specifically recognizes several RNAs, including the leader 3' *IGF2 *mRNA and *MYC *mRNAs [42-44]. *de novo *CRD-BP/IMP1 expression has been detected in human tumors of different origins, and in some of these tumors characterizes the vast majority of the samples studied (see [42,43] for references). However, the possibility of mir-483 to form triplex structures (RNA-DNA-DNA or DNA-DNA-DNA) and the possibility of CRD-BP/IMP1 protein to interact with mRNA of *IGF2 *gene might play an important role in our understanding of the specific control of *IGF2 *and mir-483 precursor expression.

## Conclusion

*TTS mapping *provides a comprehensive visual and analytical tool to help a user to find pTTSs, G4s and other regulatory DNA structures and genomic regions. Our pipeline allows us (i) to discover diverse biologically meaningful complex genome elements, (ii) identify novel biomarkers of complex diseases and their unique/specific combinations.

*TTS Mapping *provides not only bioinformatics support, due to its sequence visualization tools and statistical tables, but also integrates knowledge about many diverse DNA structures and their annotation tracks. Our results suggest that recombination-prone and mutation-prone genes *EVI-1 *and *MYC *is significantly enriched with co-occurring regulatory DNA sequences including TTSs and G4s, which could be used to develop novel approaches for gene therapy based on highly-specific TFO-TTS interactions.

*TTS Mapping *predicts the existence of a sub-set of natural ncRNAs forming hairpin secondary structures, including high-complementary and evolutionarily conserved polypurine- and polypyrimidine-rich stem and non-conserved polypurine/polypyrimidine hairpin loops with varying purine/pyrimidine content. Such R-Y paired ncRNAs could form siRNA and miRNAs, which might be involved in silencing and activation of expression of many dozens of essential genes. The DNA precursors of such Y-R paired ncRNAs might be also considered as prospective targets for high-specific anti-gene therapy.

## Methods

### Data sources

The human genome sequences are prepared for TTSs searching in two different data sets: the first data set is human genome sequence hg18 (NCBI Build 36.1); the second data set corresponds to the same genome sequence (hg18), for which DNA repeat regions were masked (RepeatMasker/RepBase update 9.11) [45]. The genome data for BLAST was prepared Makeblastdb software (NCBI Blast 2.2.19+ package) from human genome sequence without repeat masking. NCBI BlastN software [46] was used to verify the uniqueness of each TTS. UCSC annotation tracks (refGene, cpgIslandExt, snp129, targetscans, tfbsConsSites, and wgrna) were integrated for mapping pTTSs with annotation tracks. G-quadruplex sequences, predicted by Quadparser, were provided at http://www.quadruplex.org. The search engine of *TTS mapping *is available for a public access at http://ggeda.bii.a-star.edu.sg/~piroonj/TTS_mapping/TTS_mapping.php.

### Construction and Implementation of *TTSs mapping*

The aim of *TTSs mapping *pipeline is to provide users with a flexible pTTSs search tool for polypurine sequences in the human genome and to integrate the pTTSs mapping data with available annotation tracks which could be associated with structural and functional properties of the TTSs. Our web tool requests a chromosome position as an initial search parameter. To form the triplex, the given pTTS should match the following specifications [15,23,30]: (a) minimal and maximal pTTS sequence length, (b) minimal guanine (G) content percent, (c) number of non-contiguous pyrimidine insertions, and (d) human genome sequences with or without repeat masking. Detailed information about optimal selection of these parameters is presented in the next section. *TTS mapping *provides optimal parameters of pTTS searching by default and also it allows the users to choose the parameters for mapping pTTSs optionally (see section TTS search parameters in Results). Chromosome co-localization of G-quadruplexs, miRNAs, snoRNAs, miRNA gene targets, TFBSs, SNPs, nucleosome occupancy profiles could be also observed and statistically analysed. The data flow schema is showed in Figure 2. The results of the search are reported in a summary table, and are used as input data for integrate mapping of pTTSs with available annotation information.

Figure 3 shows the interface page of TTS mapping tools. Figure 4 shows how search results are combined in summary tables containing: genome region-specific information, pTTS searching parameters, statistics of pTTSs found in the given region, references to gene sequence databases (via refGene from UCSC). That information is reported for the each gene found in the given genome region overlapped with pTTS, and the annotation tracks found in the given region and overlapped with pTTSs. In addition, the summary results page provides links to view the pTTSs details and to display all the available tracks in UCSC bioinformatics browser. TTS mapping tool is implemented in Perl language.

### Criteria of identification of pTTS sequences

The *TTS mapping *tool is used to identify pTTSs in the reference human genome (hg18). The initial parameters for the program search were the following: minimal length of TTS is ≥15 nt, minimal guanine content is ≥50%, the number of pyrimidine insertions could be ranged from 0 nt to 3 nt (1 nt insertion is by default). Using these criteria, the pTTS sequences were computationally mapped on the human chromosomes. We used NCBI BLAST program for alignment of these sequences against human genome to count the copy number of the given pTTS present in the genome. The unique pTTSs defined as the sequences found only in a single position in the genome were also identified. This set of unique pTTSs was divided into three groups: short-length TTSs (15-18 nt long), medium-length TTSs (19-24 nt long), and large-length TTSs (>25 nt long). The number of pTTS in the each set was computed and compared with the number of unique pTTSs previously described by Wu et al. [22].

## Competing interests

The authors declare that they have no competing interests.

## Authors' contributions

The concept of this project was suggested by VAK. PIJ and VAK developed the TTS mapping algorithm. PIJ wrote the code of the program and maintains the relevant databases. The manuscript was discussed and written by VAK and PIJ.

## Note

Other papers from the meeting have been published as part of *BMC Bioinformatics *Volume 10 Supplement 15, 2009: Eighth International Conference on Bioinformatics (InCoB2009): Bioinformatics, available online at http://www.biomedcentral.com/1471-2105/10?issue=S15.

## Supplementary Material

Additional file 1 **The distribution of the number of pTTSs duplication in the human genome**. Blue dash line: the distribution of the number of pTTSs duplication of all pTTSs length. Other lines: the distributions of the number of pTTSs duplication of length 15 nt, 16 nt, 17 nt, 18 nt, and 19 nt, respectively. Click here for file

Additional file 2 **The comparison of the numbers of unique pTTSs counted within the groups of short, medium and long sequence lengths. TTSs with all observed lengths found in the TTS databases were counted**. The table shows a comparison of three groups of TTS length (short pTTSs, medium pTTSs, and large pTTSs). The percentage of unique pTTS versus the total number of pTTSs found in DB was calculated. Click here for file

Additional file 3 **Mapped pTTSs found in selected region (chr8:128815498-128824855)**. The list of pTTSs consists of TTS IDs, descriptive information (sequence context, length, strand, percentage of guanine, number of pyrimidine insertions, and presence of tandem repeat(s) and overlapped annotation tracks. Click here for file

Additional file 4 **The mapped pTTSs found in gene region (NM_002467.3; *MYC *gene)**. The list of pTTSs consists of TTS IDs, descriptive information (sequence context, length, strand, percentage of guanine, number of pyrimidine insertions, and presence of tandem repeat(s)), a distance between a given pTTS and Transcription Start Site (TSS), a distances between pTTS and start of gene or end of gene, and TTS gene location. Click here for file

Additional file 5 **The mapped pTTS found in the region (chr3:170281981-170350216)**. The list of pTTSs consists of TTS IDs, descriptive information (sequence context, length, strand, percentage of guanine, number of pyrimidine insertions, and presence of tandem repeat(s) and overlapped annotation tracks. Click here for file

Additional file 6 **The mapped pTTSs found in gene region (NM_001105078.2; *EVI-1 *gene)**. The list of pTTSs consists of TTS IDs, descriptive information (sequence context, length, strand, percentage of guanine, number of pyrimidine insertions, and presence of tandem repeat(s)), a distance between a given pTTS and Transcription Start Site (TSS), a distances between pTTS and start of gene or end of gene, and TTS gene location. Click here for file

Additional file 7 **The mapped list of pTTSs found in gene region (NM_001105077.2; *EVI-1 *gene)**. The list of pTTSs consists of TTS IDs, descriptive information (sequence context, length, strand, percentage of guanine, number of pyrimidine insertions, and presence of tandem repeat(s)), a distance between a given pTTS and Transcription Start Site (TSS), a distances between pTTS and start of gene or end of gene, and TTS gene location. Click here for file

Additional file 8 **The mapped list of pTTSs found in gene region (NM_005241.2; *EVI-1 *gene)**. The list of pTTSs consists of TTS IDs, descriptive information (sequence context, length, strand, percentage of guanine, number of pyrimidine insertions, and presence of tandem repeat(s)), a distance between a given pTTS and Transcription Start Site (TSS), a distances between pTTS and start of gene or end of gene, and TTS gene location. Click here for file
