# Supplementary figures and images for: TTS Mapping: integrative WEB tool for analysis of triplex formation target DNA Sequences, G-quadruplets and non-protein coding regulatory DNA elements in the human genome
Source: BMC Genomics. 2009 Dec 3;10(Suppl 3):S9. doi: 10.1186/1471-2164-10-S3-S9 (PMC2788396; doi:10.1186/1471-2164-10-S3-S9)

## TTS duplication in human genome

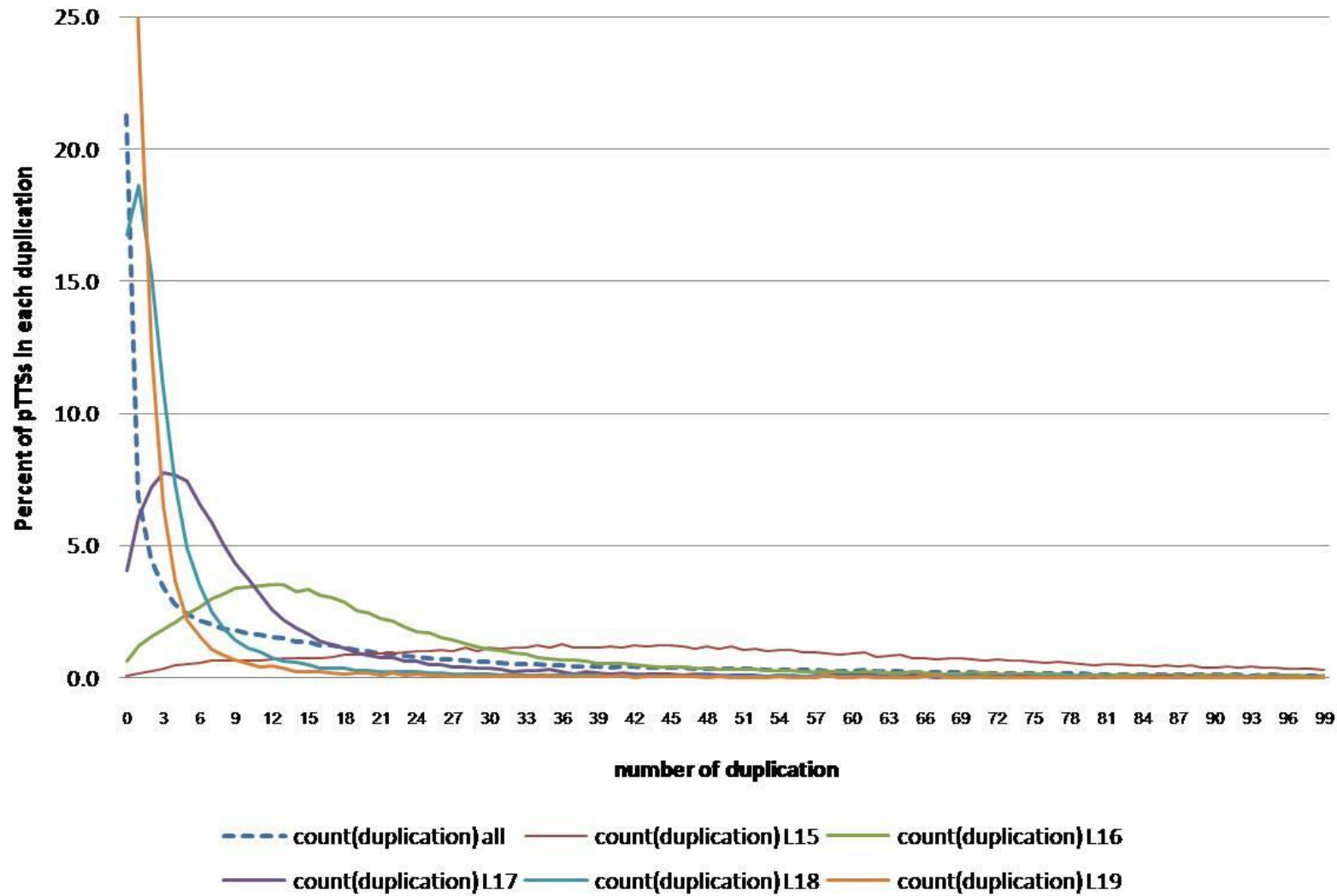

Supplement: Additional file 1 — The distribution of the number of pTTSs duplication in the human genome. Blue dash line: the distribution of the number of pTTSs duplication of all pTTSs length. Other lines: the distributions of the number of pTTSs duplication of length 15 nt, 16 nt, 17 nt, 18 nt, and 19 nt, respectively. [file 1471-2164-10-S3-S9-S1.pdf]
